# Can AMP induce sputum eosinophils, even in subjects with complete asthma remission?

**DOI:** 10.1186/1465-9921-11-106

**Published:** 2010-08-02

**Authors:** Franke Volbeda, Nick HT ten Hacken, Monique E Lodewijk, Antoon Dijkstra, Machteld N Hylkema, M Broekema, Wim Timens, Dirkje S Postma

**Affiliations:** 1 Department of Pulmonology, University Medical Center Groningen, University of Groningen, The Netherlands; 2 Department of Pathology, University Medical Center Groningen, University of Groningen, The Netherlands; 3 Pulmonary rehabilitation center Beatrixoord, University Medical Center Groningen, University of Groningen, The Netherlands

## Abstract

**Background:**

The definition of **"**clinical asthma remission" is based on absence of symptoms and use of medication. However, in the majority of these subjects airway inflammation is still present when measured. In the present study we investigated whether "complete asthma remission", additionally defined by the absence of bronchial hyperresponsiveness (BHR) and the presence of a normal lung function, is associated with the absence of airway inflammation.

**Methods:**

Patients with a former diagnosis of asthma and a positive histamine provocation test were re-examined to identify subjects with complete asthma remission (no asthma symptoms or medication, PC_20 _histamine > 32 mg/ml, FEV_1 _> 90% predicted). Patients with PC_20 _histamine ≤ 32 mg/ml were defined as current asthmatics and were divided in two groups, i.e. asthmatics with and without BHR to adenosine 5'monophoshate (AMP). Sputum induction was performed 1 week before and 1 hour after AMP provocation. Sputum induction and AMP provocation were previously shown to be sensitive markers of airway inflammation.

**Results:**

Seven patients met criteria for complete asthma remission. Twenty-three were current asthmatics, including twelve without hyperresponsiveness to AMP. Subjects with complete asthma remission showed no AMP-induced sputum eosinophilia (median (range) 0.2 (0 - 4.6)% at baseline and 0.2 (0 - 2.6)% after AMP). After AMP, current asthmatics had a significant increase in sputum eosinophils (0.5 (0 - 26.0)% at baseline and 2.6 (0 - 32.0) % after AMP), as had the subgroup of current asthmatics without hyperresponsiveness to AMP (0.2 (0 - 1.8)% at baseline and 1.3 (0 - 6.3)% after AMP).

**Conclusions:**

Subjects with complete asthma remission, in contrast to subjects with current asthma, do not respond with eosinophilic inflammation in sputum after AMP provocations. These data lend support to the usefulness of the definition of complete asthma remission.

## Background

Asthma symptoms may diminish over time in a subgroup of patients and sometimes even disappear completely. The loss of asthma symptoms in the absence of need for pulmonary medication has been defined as "clinical or symptomatic asthma remission"[1-4]. However, the majority of patients with clinical or symptomatic remission still shows bronchial hyperresponsiveness (BHR) [1-4], suggesting that the disease asthma is not yet cured.

This is supported by the observation that the broncho alveolar lavage (BAL) fluid contains increased levels of eosinophils in some asthmatic children with clinical asthma remission, when compared to healthy controls[5]. Furthermore, higher numbers of eosinophils, T cells, mast cells and expression of IL-5 were found in the airway mucosa of patients with clinical remission than in healthy controls[6].

Given the persistent presence of airway inflammation and BHR despite the asymptomatic status, it is likely that a more strict definition is required to signify whether an individual really is "cured" from asthma and can be regarded to have *complete remission*. The definition of clinical asthma remission has therefore previously been extended to "complete asthma remission", also including absence of BHR and normal lung function (FEV_1_>90%predicted) [1,4]. A 25-year follow up study of 181 asthma patients, initially aged 13-44 years, all diagnosed with a positive bronchial hyperresponsiveness test to histamine and clinical asthma symptoms according to ATS criteria, showed a prevalence of clinical remission and complete remission of 40% and 11% respectively by the age of 38-69 years[1]. In another follow up study, 119 allergic asthmatic children were retested after 30 years and 52% were in clinical remission and 22% in complete asthma remission[4]. To our knowledge, it has not been investigated so far whether asthmatic airway inflammation is objectively absent in this subgroup of subjects with complete asthma remission.

We have previously shown that adenosine 5'monophosphate (AMP) provocation induces sputum eosinophilia in asthma patients with and without inhaled steroid use[7]. We considered this as an interesting tool to investigate airway inflammation, because the recruitment process of eosinophils after an AMP challenge indicates the presence of an active inflammatory response.

The present study was set up to investigate whether such an inflammatory response indeed is absent and cannot be induced in subjects with complete asthma remission. To this aim we compared inflammatory cells 1 week before and 1 hour after AMP provocation in induced sputum from subjects with complete remission and from patients with current asthma. Since not all asthmatics are responsive to AMP, we divided these subjects in an AMP responsive and a non-responsive group. These subgroups enabled us to additionally study the dose-response effect op AMP on sputum eosinophil numbers.

## Methods

### Subjects

Non-smoking asthma patients aged between 18 and 70 years, without oral or inhaled corticosteroids were recruited. All patients originated from research cohorts investigated earlier by our research group and all had a doctor's diagnosis of asthma and a documented PC_20 _histamine ≤ 32 mg/ml in the past[4,8-10]. All patients had to be able to expectorate sputum after inhalation of hypertonic saline. In order to compare the effects of the highest cumulative dose of AMP (639.99 mg) on the influx of inflammatory cells in induced sputum, current asthma patients were divided in those with a negative and a positive AMP provocation test. Current asthma in these patients was proven by a positive bronchial hyperresponsiveness test to histamine. Patients were considered to have no asthma symptoms when they answered negatively on questions regarding cough and sputum in wintertime, dyspnea, wheeze and asthma attacks in the last three years. The study protocol was approved by the local medical ethics committee; all participants gave their written informed consent.

Patients were assigned to 3 different groups according to the following criteria:

• *Complete asthma remission*: former diagnosis of asthma, PC_20 _AMP > 320 mg/ml and PC_20 _histamine > 32 mg/ml, FEV_1 _% predicted > 90%, no asthma symptoms, no asthma medication.

• *Current asthma with a negative PC_20 _AMP*: former diagnosis of asthma, PC_20 _AMP > 320 mg/ml, PC_20 _histamine ≤ 32 mg/ml.

• *Current asthma with a positive PC_20 _AMP*: former diagnosis of asthma, PC_20 _AMP ≤ 320 mg/ml.

### Study Design

Patients visited the hospital twice. At the first visit lung function, blood collection and sputum induction were obtained. The second visit followed after 1-2 weeks and included AMP provocation test and sputum induction 1 hour after the final dose of AMP. In patients with a negative AMP provocation (dose AMP > 320 mg/ml) bronchial hyperresponsiveness to histamine was measured > 1 week later. To facilitate comparison with historical data on bronchial hyperresponsiveness to histamine, the maximum provocative dose of histamine in the present study was also set at 32 mg/ml (with 30-seconds tidal breathing method, a dose that is comparable with 8 mg/ml in the 2-minute tidal breathing method).

### Questionnaire

The Dutch version of the British Medical Research Council's standard questionnaire was used[11]. Patients were considered asymptomatic if they answered negatively on questions regarding cough, sputum, dyspnea, wheeze and asthma attacks

### Lung Function

FEV_1 _was measured with a calibrated water-sealed spirometer according to standardized guidelines[12,13]. Reversibility of the FEV_1_%predicted was measured after administration of 400 μg of salbutamol. Provocation tests were performed with a method adapted from Cockcroft and coworkers[14,15]. After 2-min tidal breathing and an initial nebulized saline challenge, subjects inhaled doubling concentrations AMP (0.04 to 320 mg/ml) at 5-min intervals. Bronchial hyperresponsiveness to histamine was tested as reported previously[8], using 30-seconds tidal breathing and doubling concentrations ranging from 0.13 to 32 mg/ml.

### Sputum Induction and Sputum Processing

Sputum was induced by inhalation of hypertonic saline aerosols as previously described[13]. Hypertonic saline (5%) was nebulized over 3 consecutive periods of 5 min. Whole sputum samples were processed according to the method of Fahy and colleagues with some modifications[13,16]. Sputum cell cytospins were stained with May Grünwald Giemsa (MGG) and cell differentials from in total 600 viable, non squamous cells were assessed in a blinded fashion. Sputum was not scored if the percentage of squamous cells was > 80 percent or the total number of non-squamous cells was < 600. Number of sputum drop-outs because of 80% squamous cells were 6 at baseline and 0 after AMP, drop outs because of < 600 non-squamous cells were 2 at baseline and 4 after AMP. Additionally, no sputum could be induced in 8 patients at baseline and 6 after AMP.

### Histamine ELISA

A histamine ELISA was purchased from IBL (Hamburg, Germany) for quantitative detection of histamine in sputum supernatant. All reagents were provided in the kit. The protocol was as follows: the acetylated samples, controls and standards are pipetted into a 96-wells plate. After adding enzyme conjugate and histamine antiserum the plate was incubated for 3 hours on an orbital shaker. After washing TMB substrate solution was added to each well and incubated for 20 minutes. The substrate reaction was stopped and the optical density was measured at 450 nM.

### Allergic Parameters

The concentration of eosinophilic cationic protein (ECP) in sputum was measured using a fluoroenzyme immunoassay (ImmunoCAP ECP, Pharmacia, Uppsala, Sweden). Total serum IgE (IU/L) was measured by a solid-phase immunoassay (VIDAS total IgE kit, BioMérieux, Marcy l'Etoile, France). The Phadiatop screening test was performed on the ImmunoCap system according to the instructions of the manufacturer (Phadia AB, Sweden). Results were presented as quotients (fluorescence of the serum of interest divided by the fluorescence of a control serum). Positive Phadiatop was defined as patient serum/control serum >1.

### Statistics

All analyses were performed using SPSS (version 16.0; SPSS Inc, Chicago, IL, USA). Non-parametric tests were used for analysis. Wilcoxon signed rank test was used for paired testing within groups between measurements at baseline and after AMP provocation. Mann-Whitney U test was used for testing differences between groups. Two tailed p-values of < 0.05 were considered statistically significant.

## Results

### Patient Characteristics

47 patients were enrolled in the study. 17 patients were excluded from analysis because they were not able to produce sputum both before and after AMP provocation or because the quality of sputum was too low to allow analysis. 3 of the excluded subjects had complete asthma remission and 14 current asthma. The included and excluded groups did not differ statistically regarding age, sex, FEV_1 _%pred, reversibility of FEV_1_, allergy and blood eosinophils. From the included patients, 7 patients met the inclusion criteria for complete asthma remission and were compared with 23 current asthma patients. Of these current asthmatics 11 had a positive PC_20_AMP (PC_20 _AMP ≤ 320 mg/ml) and 12 had a negative PC_20_AMP (PC_20 _AMP > 320 mg/ml, but a PC_20 _Histamine ≤ 32 mg/ml) (table 1). FEV_1 _% predicted in subjects with complete asthma remission was significantly higher than in patients with current asthma. Atopy was most frequent in asthmatics with a positive PC_20_AMP, significantly more frequent than in current asthmatics with a negative PC_20_AMP and subjects in complete asthma remission

**Table 1:** Patient Characteristics

	Complete remission	Current asthma		
		
	(n = 7)	Total group (n = 23)	Negative PC_20 _AMP (n = 12)	Positive PC_20 _AMP (n = 11)
Sex (M/F)	2/5	14/9	6/6	8/3
Age (years)	53.0 (32 - 67)	48.0 (35 - 70)	47.5 (35 - 70)	48.0 (38 - 62)
FEV_1 _(% predicted)	108.0 (102 - 144)*,^‡‡^	97 (76 - 131)	96.2 (76.2 - 123)	96.5 (84.8 - 131)
PC_20 _AMP (mg/ml)	>320	>320 (0.02 - >320)	>320	12.0 (0.02 - 174)
Cumulative AMP dose (mg)	639.99	639.99 (0.02 - 639.99)	639.99	39.99 (0.02 - 639.99)
PC_20 _Histamine (mg/ml)	>32.0	N.A.	16.9 (2.5 - 32.0)	N.A.
Reversibility FEV_1 _(%)	4.59 (1.76 - 9.72)	5.42 (-2.98 - 26.7)	3.15 (-2.98 - 12.2)^‡^	9.40 (1.76 - 26.7)
IgE (IU/L)	47 (7 - 166)	66 (11 - 558)	61 (14 - 246)	78 (11 - 558)
Positive Phadiatop (n (%))	3 (43)^‡^	15 (65)	5 (42)^‡^	10 (91)
Eosinophils blood (× 10^9^/L)	0.12 (0.09 - 0.24)	0.15 (0.03 - 0.35)	0.11 (0.03 - 0.35)	0.17 (0.07 - 0.25)

Values are medians (ranges), unless stated otherwise.

*Definition of abbreviations*: PC_20 _AMP = provocative concentration of adenosine 5'monophoshate causing a 20% fall in FEV_1; _reversibility FEV_1 _= change in FEV_1_, expressed as increase in percentage predicted normal value after 400 μg of Salbutamol; Positive Phadiatop = specific IgE's in patient serum/control serum >1; n = number; N.A. = not available.

* p ≤ 0.05 versus current asthma total group.

^‡ ^p ≤ 0.05, ^‡‡ ^p ≤ 0.01 versus current asthma PC_20 _AMP positive.

### Sputum Data

#### Complete Asthma Remission versus Current Asthma

**Figure 1:**
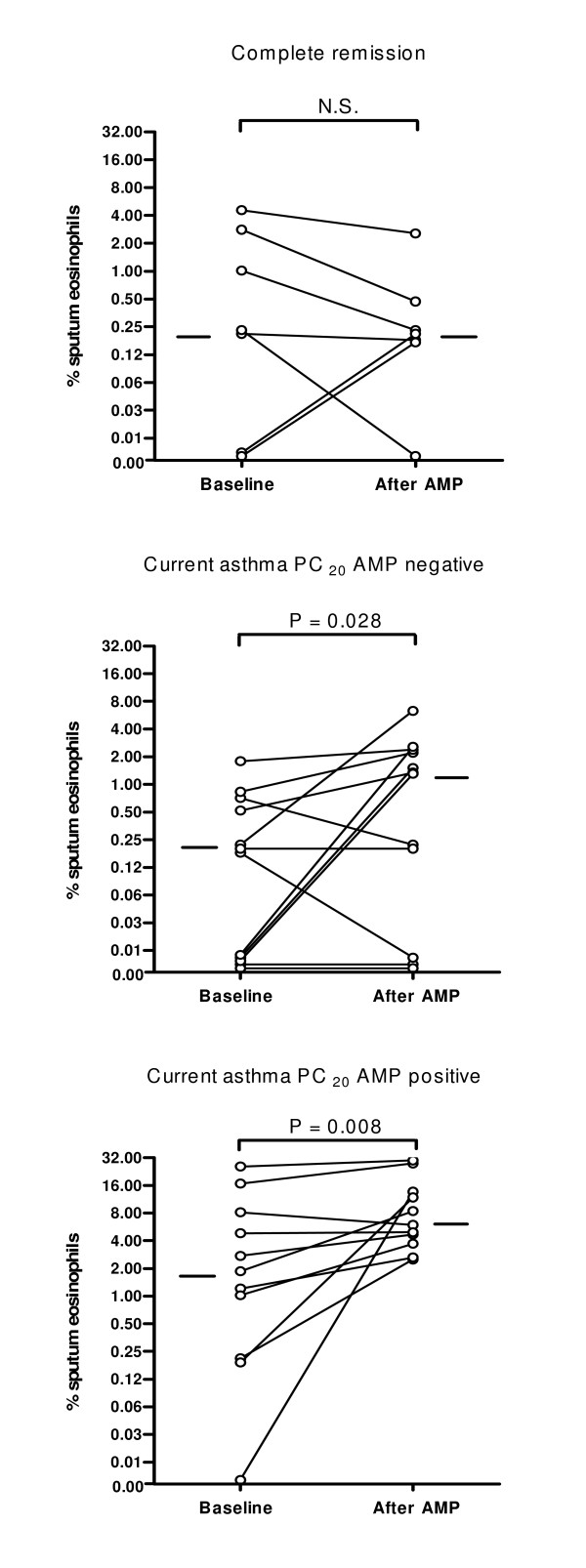
**Changes in sputum eosinophil % from baseline to post-AMP provocation in subjects with complete asthma remission, current asthma patients with a negative AMP provocation and current asthma patients with a positive AMP provocation**. Data are presented in a semi-log plot to optimize visualization of minor and major changes.

At baseline, total sputum cell count, macrophage, neutrophil, lymphocyte and eosinophil differential counts were similar in all investigated groups (table 2). After AMP provocation, sputum eosinophils did not increase in subjects with complete asthma remission in contrast to patients with current asthma, resulting in a significantly higher percentage eosinophils post AMP challenge in the current asthma groups (figure 1). Similar trends were observed for sputum ECP, i.e. median (range) levels before and after AMP were 16.0 (2.5 - 170.0) and 14.8 (2.6 - 63.7), respectively, in the group with complete asthma remission and 36.3 (6.0 - 2467.0) and 47.3 (9.0 - 1628.0) in the group with current asthma. Histamine levels in sputum supernatant were comparable in the group with complete asthma remission and with current asthma. Median (range) levels before and after AMP provocation were 13.4 (2.8 - 29.8) ng/ml and 12.4 (4.0 - 137.0) ng/ml, respectively, in the group with complete asthma remission, and 9.8 (0.2 - 38.0) ng/ml and 8.4 (3.0 - 30.5) ng/ml in the group with current asthma.

**Table 2:** Sputum before and after amp Provocation

	Complete remission	Current asthma		
		
	(n = 7)	Total group (n = 23)	Negative PC_20 _AMP (n = 12)	Positive PC_20 _AMP (n = 11)
**Total cells (×10^6^/ml)**				
Baseline	0.6 (0.1 - 2.9)	0.3 (0.1 - 2.3)	0.4 (0.1 - 2.3)	0.3 (0.2 - 2.1)
After AMP	0.2 (0.1 - 1.4) ^θ^	0.3 (0.1 - 3.0)	0.4 (0.2 - 3.0)	0.3 (0.1 - 0.8)
Change	-0.5 (-1.5 - 0.0)	0.0 (-1.7 - 1.5)	0.1 (-1.1 - 1.5)	0.0 (-1.7 - 0.2)
**Squamous cells (%)**				
Baseline	23.8 (5.3 - 67.0)	21.3 (0.8 - 70.3)	19.2 (0.8 - 70.3)	21.3 (4.7 - 43.3)
After AMP	41.5 (9.0 - 49.0)	17.5 (0.5 - 64.5)	19.9 (0.5 - 64.5)	17.5 (1.7 - 24.5)
Change	14.0 (-26.2 - 22.2)	-2.0 (-25.8 - 27.0)	-1.1 (-16.5 - 27.0)	-2.0 (-25.8 - 10.6)
**Neutrophils (%)**				
At baseline	62.2 (18.9 - 77.6)	55.2 (19.8 - 94.5)	56.8 (20.7 - 94.5)	55.2 (19.8 - 93.6)
After AMP	48.1 (15.0 - 90.4)	59.0 (22.3 - 96.8)	71.3 (25.7 - 96.8)	52.7 (22.3 - 71.0)
Change	-3.9 (-37.9 - 58.1)	-3.2 (-35.1 - 68.0)	-0.4 (-29.5 - 68.0)	-5.1 (-35.1 - 25.4)
**Macrophages (%)**				
At baseline	35.8 (19.9 - 77.7)	32.7 (5.3 - 76.4)	40.0 (5.3 - 76.4)	32.7 (5.6 - 76.2)
After AMP	49.1 (8.9 - 84.2)	29.2 (3.0 - 72.5)	23.1 (3.0 - 72.5)	39.1 (12.6 - 61.2)
Change	11.2 (-56.7 - 41.3)	1.2 (-65.1 - 28.6)	0.0 (-65.1 - 28.6)	1.2 (-37.1 - 20.6)
**Eosinophils (%)**				
At baseline	0.2 (0.0 - 4.6)	0.5 (0.0 - 25.7)	0.2 (0.0 - 1.8)^‡‡^	1.9 (0.0 - 25.7)
After AMP	0.2 (0.0- 2.6)*^, ‡‡‡^	2.6 (0.0 - 32.0)^θ θ θ^	1.3 (0.0 - 6.3)^‡‡‡, θ^	5.9 (2.5 - 32.0)^θ θ^
Change	-0.2 (-2.3 - 0.2)**^, ‡‡, §§^	1.4 (-2.3 - 13.7)	0.7 (-0.5 - 6.1)^‡^	2.7 (-2.3 - 13.7)
**Lymphocytes (%)**				
At baseline	2.2 (1.4 - 7.3)	1.9 (0.0 - 6.9)	2.0 (0.2 - 6.9)	1.9 (0.0 - 5.9)
After AMP	0.3 (0.0 - 2.5)*^, ‡‡, θ, §^	1.3 (0.0 - 6.1)	0.8 (0.0 - 6.1)	1.7 (0.7 - 4.8)
Change	-1.8 (-7.0 - 0.7)	-0.2 (-4.9 - 4.8)	-0.4 (-4.2 - 3.8)	0.1 (-4.9 - 4.8)

Squamous cells were not included in the number of total cells and in the percentages of all other cell types.

Values are medians (ranges).

* p ≤ 0.05, ** p ≤ 0.01 versus current asthma total group.

^‡ ^p ≤ 0.05, ^‡‡ ^p ≤ 0.01, ^‡‡‡ ^p ≤ 0.001 versus current asthma PC_20 _AMP positive.

^§ ^p ≤ 0.05, ^§§ ^p ≤ 0.01 versus current asthma PC_20 _AMP negative.

^θ ^p ≤ 0.05, ^θ θ ^p ≤ 0.01, ^θ θ θ ^p = 0.001 versus at baseline.

AMP provocation had little effect on the other inflammatory cells. Analysis with absolute sputum cell numbers showed comparable results with sputum cell percentages, however data are not presented because of differences in total cell numbers between groups.

#### AMP Positive versus AMP Negative Subjects with Current Asthma

Allergy was more frequently present in current asthmatics with a positive than with a negative AMP provocation test (table 1). Sputum eosinophils increased significantly after AMP provocation in current asthma patients, even in those without AMP responsiveness (figure 1). However, the increase in sputum eosinophils after AMP provocation was significantly higher in AMP positive subjects (table 2). Also higher ECP levels were observed in AMP positive current asthma patients, i.e. median levels before and after AMP were 41.6 (11.9 - 262.0) μg/l and 62.2 (15.2 - 467.0) μg/l, respectively, compared to 32.3 (6.0 - 2467.0) μg/l and 37.8 (9.0 - 1628.0) μg/l in AMP negative current asthma patients. Histamine levels in sputum supernatant were comparable in AMP positive and AMP negative subjects with current asthma. Median (range) levels before and after AMP were 11.0 (4.8 - 38.0) ng/ml and 9.1 (4.9 - 28.0) ng/ml, respectively, in the AMP positive group, and 9.5 (0.2 - 28.8) ng/ml and 8.3 (3.0 - 30.5) ng/ml in the AMP negative group.

## Discussion

To our knowledge, this is the first study investigating sputum inflammation in subjects meeting the criteria for complete asthma remission. Our definition of complete asthma remission also includes a normal lung function and absence of bronchial hyperresponsiveness in addition to the absence of asthma symptoms and medication, which was previously defined as clinical asthma remission. Sputum inflammation at baseline and the response after AMP provocation was compared between subjects with current asthma and complete asthma remission. Levels of sputum eosinophils at baseline and blood eosinophils were not significantly different between these groups. However, we demonstrate that AMP provocation increases the number of sputum eosinophils in current asthmatics, yet not in those with complete asthma remission. Similar trends were observed for ECP. This thus suggests that the latter group indeed has outgrown their asthma and eosinophils are not in a primed state.

The present study shows the usefulness of measuring hyperresponsiveness and FEV_1 _in recognizing subjects with complete asthma remission. Several studies have shown that a clinical definition based on the absence of asthma symptoms and no use of asthma medication is insufficient to definitely assess that asthma is cured. Many of these subjects still show features of persistent asthma, such as presence of hyperresponsiveness and/or a low lung function[1-4] or ongoing airway inflammation[5,6]. The concept of complete asthma remission has previously been introduced as an alternative to the definition of clinical asthma remission. Complete asthma remission is defined by the combined absence of asthma symptoms, asthma medication, airway obstruction, and bronchial hyperresponsiveness[1,4]. In the present study, we investigated the concept of complete asthma remission in more detail by measuring inflammatory cells in induced sputum before and after AMP provocation in subjects meeting the criteria for complete asthma remission. The finding that sputum eosinophils do not increase significantly after AMP provocation in these subjects supports the hypothesis that they are free of airway inflammation. Indeed, eosinopils that are characteristic of asthma are not attracted to the airways upon provocation with a stimulus that does attract these cells in patients with persistent asthma, even when similar doses of AMP are being inhaled.

This study also demonstrates that sputum induction after AMP provocation gives more information than sputum induction at baseline alone. After all, the differences between asthma remission and current asthma would not have been recognized if only baseline sputum measurements had been compared, especially since all asthma patients had stable disease without using inhaled steroids. We can speculate about the underlying mechanisms that are responsible for the differences observed. Apparently the immunological mechanisms involved in recruiting eosinophils into the airway lumen differs between subjects with complete asthma remission and patients with current asthma. It is possible that immunologically primed mast cells may have disappeared in subjects with complete asthma remission. Thus activation of the adenosine 2b receptor by AMP might not take place and neither the release of mast cell mediators. The loss of immunologically primed mast cells was not reflected by our data on histamine levels in sputum supernatant, histamine levels being similar in all groups. Bronchial biopsy studies are needed to further investigate the role of mast cells in complete asthma remission.

This study does not completely prove that our subjects with complete asthma remission fully have outgrown their asthma, as we did not compare results with healthy controls. Several studies suggest that a cut off value of 1% for sputum eosinophils can discriminate between current asthma and healthy controls[17-19]. In our study, two subjects with complete asthma remission had > 1% sputum eosinophils, possibly indicating that asthmatic inflammation may still be present in the airways. However, only one subject in the complete asthma remission group had a sputum eosinophil percentage > 1% after AMP provocation. To our knowledge, no studies have investigated AMP induced sputum eosinophils in healthy controls, which clearly needs further study. Obviously, biopsy studies comparing subjects with complete asthma remission and healthy controls are needed to obtain a definite answer as to whether airway inflammation has disappeared entirely in subjects with complete asthma remission. Such studies might validate sputum induction after AMP provocation as a useful non-invasive tool to recognize patients who really have outgrown their asthma. Also longitudinal studies are needed to investigate if symptoms do not recur in patients suspected to be in complete asthma remission.

To compare the effects of the high cumulative doses of AMP on the influx of sputum inflammatory cells, we divided patients with current asthma in groups with and without a bronchoconstrictive response to AMP. The PC_20 _AMP negative group, that still had a proven bronchoconstrictive response to histamine, received exactly the same cumulative dose of AMP as the complete asthma remission group (with negative PC_20 _histamine). In contrast to subjects with complete asthma remission, this group of current asthma patients with a negative PC_20 _AMP showed a modest but significant increase in sputum eosinophils after AMP provocation. This result indicates that AMP has a differential effect on bronchoconstriction and bronchial inflammation in asthmatics with proven hyperresponsiveness to histamine.

An intriguing finding is the discrepancy between the presence of a positive PC_20 _histamine and absence of PC_20 _AMP in current asthmatics. We realize that the cut off values of 320 mg for PC_20 _AMP and 32 mg/ml for histamine are arbitrary and not objectively based on the presence or absence of asthmatic airway inflammation. Nevertheless, it may well be that current asthmatics with a positive PC_20 _AMP represent a different subset of asthma patients. Indeed, significant differences between the two groups are present in our study. Despite a lower cumulative dose of AMP, those with PC_20 _AMP had significantly more sputum eosinophilia than those without a PC_20 _AMP and similar trends were observed with sputum ECP. In addition, reversibility to salbutamol and the presence of specific IgE in serum were significantly higher in the asthma group with a positive PC_20 _AMP. A similar association between AMP sensitivity and atopic status has been described in an earlier study[20]. It would be of interest to follow the asthmatics with a negative AMP test to assess whether they will develop clinical asthma remission. In other words it might represent an intermediate state between full blown asthmatic inflammation and absence of inflammation.

## Conclusions

We conclude that our results suggest that the definition of complete asthma remission is valid, since sputum eosinophils do not increase after AMP provocation. This is in contrast with the increase in sputum eosinophils in current asthma that occurs even in the absence of a bronchoconstrictive response to AMP.

## Competing interests

The authors declare that they have no competing interests.

## Authors' contributions

FV collected data (patient inclusion), performed statistic analyses, and wrote the article. NtH was involved in study design and setup, collected data (biopsy), assisted in analyses, writing, and revising of the article. ML processed data (sputum analyses), assisted in writing. AD collected data (patient inclusion), assisted in writing. MH assisted in analysis, writing, and revising of the article. MB assisted in writing, and revising of the article. WT was involved in study design and setup, supervised sputum data processing, assisted in analysis, writing, and revising of the article. DP was involved in study design and setup, supervised patient inclusion, assisted in analysis, writing, revising and gave final approval to the article.
